# “Safe Care for Every Newborn and Child”: Patient Safety as a Priority from the Start

**DOI:** 10.1590/1518-8345.0000.4659

**Published:** 2025-06-02

**Authors:** Mavilde Luz Gonçalves Pedreira, Aline Santa Cruz Belela-Anacleto

**Affiliations:** 1 Universidade Federal de São Paulo, Escola Paulista de Enfermagem, São Paulo, SP, Brazil

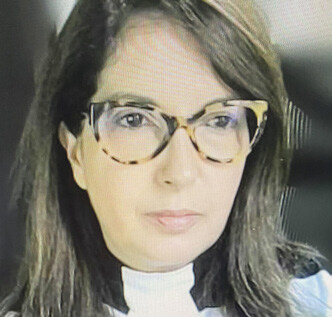

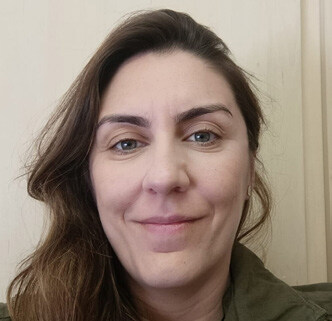





To raise global awareness and promote recognition of the need to prioritize and address the vulnerability of children to risks and harm associated with unsafe healthcare, the World Health Organization (WHO) has announced “Safe Care for Every Newborn and Child” as the theme for World Patient Safety Day 2025, celebrated on September 17 - a key date in the global public health calendar^(1)^.

The WHO urges member states to implement policies and interventions focused on priority areas to “increase global awareness of safety risks in pediatric and neonatal care across all healthcare settings, emphasizing the specific needs of children, families, and caregivers; mobilize governments, health organizations, professional bodies, and civil society to adopt sustainable strategies for safer newborn and child care, integrated into broader patient safety and quality initiatives; empower parents, caregivers, and children through education, awareness, and active participation in care and advocate for strengthened research on patient safety in pediatric and neonatal care^(1)^”.

As pediatric nurses, we welcome this initiative with enthusiasm and a sense of responsibility, recognizing its potential to align healthcare with the safety demands of newborns, children, adolescents, and their families in our country. Children’s vulnerability and heightened risk of harm from adverse events - due to demographic, developmental, and dependency factors, as well as distinct disease epidemiology (the “4 Ds of pediatrics”) - make their prioritization in patient safety policies imperative^(2)^.

Children are disproportionately affected by adverse events linked to socioeconomic disadvantage, environmental hazards, lack of access to care (especially for congenital and critical conditions), and disparities tied to age, race, ethnicity, and gender^(2-3)^. A stark example is the lack of age-appropriate medications, which elevates risks of harm and even death due to medication errors - a pervasive issue in settings ranging from homes to intensive care units. Despite progress, the availability of pediatric- and neonatal-specific drug formulations remains limited, complicating practice and increasing error susceptibility. In Brazil, unlike other countries, the commercialization of pediatric drug formulations (particularly parenteral ones, widely used for decades) is not yet mandatory. Addressing this age-based inequity requires targeted studies and/or the inclusion of children in clinical trials.

Medication safety is a cornerstone of pediatric patient safety. Though often underestimated, medication errors remain a significant cause of harm in this population. Hospitalized children face up to three times higher rates of adverse drug events than adults, with neonates at particularly high risk. Factors such as physiological immaturity, rapid developmental changes, and the need for individualized dosing (involving complex calculations and multiple prescription, preparation, and administration variables) heighten this risk^(4)^.

Experts assert that medication safety in pediatrics has long been neglected. Urgent steps include implementing tools to monitor the frequency and severity of drug-related adverse events in routine care, enabling targeted prevention strategies. Concurrently, leveraging clinical decision-support technologies - for allergy alerts, drug interactions, dosing, and clinical conditions - is critical. For neonates, research must address their unique ethical, practical, and welfare considerations^(5)^.

Child development is a dynamic, complex process that underpins all healthcare actions aimed at fostering children’s physical, mental, emotional, and social well-being. Thus, pediatric care demands expertise in developmental stages and the diverse diseases affecting this age group. Even within a single care setting, comprehensive - yet highly specialized - knowledge is essential to manage respiratory, cardiac, neurological, gastrointestinal, genitourinary, dermatological, orthopedic, and other conditions. The WHO highlights diagnostic safety concerns, as delayed or incorrect diagnoses in children often lead to severe or irreversible harm. Moreover, children’s dependency on adults - especially the very young - adds complexity: they cannot make decisions, control treatments, question care, or fully articulate their medical history. This underscores the need for professionals skilled in family engagement and strategies to educate children, adolescents, and families as active agents in patient safety^(2-3)^.

Global epidemiological studies demonstrate that nursing staffing levels and qualifications directly influence patient mortality and morbidity. Imbalances between care demands and adequately trained nurse supply compromise patient safety and public trust in healthcare systems. For children, evidence shows that nurse qualification - more than numbers alone - reduces adverse events^(6)^. Their vulnerability demands higher nurse-to-patient ratios; some U.S. states, for example, mandate ratios of up to four children per bachelor’s-prepared nurse in clinical/surgical units and one nurse per ICU bed.

In Brazil, advancing pediatric patient safety requires policies that improve both nursing workforce numbers and education quality. Nursing curricula vary widely, with neonatology sometimes treated as a specialty rather than a core competency. This approach jeopardizes safety for an already high-risk group and perpetuates social injustice by neglecting that child development must be taught as a fundamental aspect of holistic human care.

Progress demands recognizing children’s right to participate in care decisions. Information must be shared in age-appropriate ways, with their voices heard, included, and respected—ensuring care aligns with their best interests. This model acknowledges children as active, capable social agents whose needs and perspectives should shape health interventions^(7)^.

We hope issues promoting safety for all newborns and children will become national research priorities, strengthening Brazil’s Unified Health System (SUS) and advancing sustainable development.

As citizens, children have a right to healthcare, and society has a duty to protect them. As nurses, we are ethically bound to provide safe, competent, and dignified care - free from preventable harm - upholding their right to “Patient Safety from the Start”.
