# Supplementary material for: “Safe Care for Every Newborn and Child”: Patient Safety as a Priority from the Start
Source: Rev Lat Am Enfermagem. 2025 Jun 2;33:e4659. doi: 10.1590/1518-8345.0000.4659 (PMC12129444; doi:10.1590/1518-8345.0000.4659)
Supplement: Translation

# Translation: “Cuidado seguro para todo recém-nascido e toda criança”: a segurança do paciente como prioridade desde o início

**DOI:** 10.1590/1518-8345.0000.4660

**Published:** 2025

**Authors:** Mavilde Luz Gonçalves Pedreira, Aline Santa Cruz Belela-Anacleto

**Affiliations:** 1 Universidade Federal de São Paulo, Escola Paulista de Enfermagem, São Paulo, SP, Brasil





Com o objetivo de sensibilizar e promover a conscientização global sobre a necessidade de reconhecer, priorizar e abordar a vulnerabilidade das crianças diante dos riscos e danos associados à prestação insegura de assistência à saúde, a Organização Mundial da Saúde (OMS) anunciou “Cuidado seguro para todo recém-nascido e toda criança” como tema do Dia Mundial da Segurança do Paciente de 2025, celebrado em 17 de setembro, data relevante no calendário global de saúde pública da entidade^(1)^.

A OMS incentiva a ação dos países membros na implementação de políticas e intervenções focadas em áreas prioritárias, para “aumentar a conscientização global sobre os riscos de segurança no cuidado pediátrico e neonatal em todos os ambientes de saúde, enfatizando as necessidades específicas de crianças, famílias e cuidadores; mobilizar governos, organizações de saúde, entidades profissionais e a sociedade civil para implementar estratégias sustentáveis para um cuidado mais seguro para recém-nascidos e crianças, como parte de iniciativas mais amplas de segurança do paciente e qualidade; capacitar pais, cuidadores e crianças em segurança do paciente, promovendo educação, conscientização e participação ativa no cuidado e advogar pelo fortalecimento da pesquisa em segurança do paciente no cuidado pediátrico e neonatal”^(1)^.

Na identificação desta iniciativa, enquanto enfermeiras pediatras, expressamos nosso entusiasmo e senso de responsabilidade ao reconhecermos a relevância e o potencial impacto dessa ação na promoção de cuidados de saúde mais alinhados às demandas de segurança para neonatos, crianças, adolescentes e suas famílias em nosso país. A necessidade de prioridade das crianças nas políticas de segurança do paciente decorre de sua vulnerabilidade e do maior risco de danos decorrentes de eventos adversos por fatores demográficos, de desenvolvimento, dependência dos pais ou outros cuidadores e das diferentes epidemiologias das doenças, conhecidos como os 4Ds da pediatria^(2)^.

Crianças apresentam uma maior propensão a sofrer eventos adversos associados a condições socioeconômicas desfavoráveis, sendo mais suscetíveis a problemas sanitários e ambientais, ao risco de falta de atendimento, particularmente para o tratamento de agravos congênitos e doenças críticas, além das desigualdades no acesso aos cuidados, que envolvem questões relacionadas à idade, cor da pele, etnia e gênero^(2-3)^.

Um exemplo claro é a falta de acesso a medicamentos adequados para cada faixa etária, o que acarreta elevados riscos de danos e até mortes associados a erros de medicação, presentes em diversas situações de cuidado de crianças, que vão desde o domicílio até a terapia intensiva. Embora tenham ocorrido avanços nas últimas décadas, a disponibilidade de medicamentos com características e formulações específicas para uso pediátrico e neonatal ainda é limitada, tornando essa prática mais complexa e suscetível a falhas. No Brasil, a comercialização de apresentações pediátricas de medicamentos, especialmente os de uso parenteral, amplamente utilizados na prática assistencial há décadas, ainda não é obrigatória, como já ocorre em outros países. Assim, é fundamental destacar a importância de enfrentar o desafio de combater a desigualdade etária nessa área, com a realização de estudos específicos e/ou a inclusão de crianças em ensaios clínicos com medicamentos.

A terapia medicamentosa representa uma das temáticas prioritárias no contexto da segurança do paciente pediátrico. Ainda que sistematicamente subestimados, erros de medicação continuam a representar causa significativa de danos nessa faixa etária. Em crianças hospitalizadas, as taxas de eventos adversos relacionados a medicamentos podem ser até três vezes maiores do que em adultos, sendo que os neonatos apresentam risco particularmente elevado. A imaturidade, as rápidas modificações desenvolvimentais e fisiológicas associadas à necessidade de cálculo individualizado da dose, envolvendo múltiplas operações matemáticas e diversas variações na prescrição, no preparo e na administração dos medicamentos, são fatores que contribuem para o maior risco de ocorrência de eventos adversos^(4)^.

Especialistas afirmam que a segurança com medicamentos em pediatria representa uma área negligenciada por demasiado tempo. É urgente a necessidade de implementação de ferramentas que monitorem a frequência e a gravidade dos eventos adversos relacionados a medicamentos na rotina assistencial, subsidiando a implantação de estratégias de prevenção específicas. Paralelamente, é crucial o uso adequado de recursos tecnológicos de suporte à decisão clínica em diferentes aspectos, como identificação de alergias, interações medicamentosas, dosagem e condições clínicas. No que se refere ao neonato, são necessários estudos delineados especificamente para essa população, considerando características práticas, éticas e de bem-estar^(5)^.

O desenvolvimento infantil é um processo dinâmico e complexo, que fundamenta e orienta todas as ações de atenção à saúde, visando à promoção das capacidades plenas das crianças, assim como o seu bem-estar físico, mental, emocional e social. Portanto, a atuação nesta especialidade requer conhecimento sobre o desenvolvimento da criança, desde o nascimento até a adolescência, e sobre as diversas doenças que acometem este grupo etário. Vale ressaltar que, em um mesmo ambiente assistencial, é necessário um conhecimento abrangente e, ao mesmo tempo, específico, para uso de diferentes tecnologias e intervenções no atendimento de agravos respiratórios, cardíacos, neurológicos, gastrointestinais, geniturinários, dermatológicos, ortopédicos, entre outros. Esta situação é ressaltada pela OMS no que tange à segurança diagnóstica, pois o atraso e o erro diagnóstico em crianças geram eventos adversos graves ou de difícil reversão. Além disso, a dependência das crianças em relação aos adultos, especialmente as mais jovens, torna ainda mais complexo o cuidado, pois elas não tomam decisões sozinhas, não têm controle sobre o tratamento, não conseguem questionar o atendimento recebido nem fornecer sua história clínica, o que torna essencial contar com profissionais preparados para o engajamento da família e a promoção de estratégias para preparar e educar as crianças, os adolescentes e familiares a se tornarem agentes ativos na promoção da segurança do paciente^(2-3)^.

Neste contexto, estudos epidemiológicos abrangentes, realizados em países de todos os continentes, demonstram que tanto a quantidade quanto a qualificação dos profissionais de enfermagem influenciam diretamente os índices de mortalidade e morbidade dos pacientes. O desequilíbrio entre a demanda por cuidados e a oferta de profissionais de enfermagem, em número e qualificação adequados, compromete a segurança do paciente e a confiança da população no sistema de saúde. No cuidado de crianças, estudos evidenciam que mais importante do que o aumento do número de enfermeiros é sua qualificação, impactando diretamente na redução de eventos adversos^(6)^. A vulnerabilidade das crianças exige mais enfermeiros envolvidos na assistência, sendo recomendada em alguns estados norte-americanos, por exemplo, uma proporção de até quatro crianças por enfermeiros bacharéis em unidades de atendimento clínico e cirúrgico, e um por leito em unidades de terapia intensiva.

No contexto nacional, para promover a segurança do paciente pediátrico, é fundamental que as políticas se concentrem em melhorar tanto o número quanto a qualidade da formação dos profissionais de enfermagem. No ensino superior, os currículos da enfermagem pediátrica apresentam uma grande variação e, em algumas estratégias curriculares, a neonatologia é tratada como uma especialidade. Essa abordagem compromete não apenas a segurança desse grupo etário, já altamente vulnerável durante as intervenções e cuidados em saúde, mas também a justiça social, ao desconsiderar que essa fase do desenvolvimento humano deve ser abordada de forma generalista, como parte do ensino essencial para a formação de profissionais capacitados a lidar com o processo vital humano em sua totalidade.

O avanço do cuidado seguro do paciente pediátrico requer o reconhecimento do direito da criança de participar das tomadas de decisões referentes a seu próprio cuidado. É imperativo que as informações sejam compartilhadas de forma compatível a sua idade e ao seu nível de desenvolvimento, e que elas sejam ouvidas, incluídas, protegidas e tratadas com respeito e em seu real melhor interesse. Trata-se da implementação de um modelo de cuidado que dá voz à criança, reconhecendo-a como agente social em desenvolvimento, ativo e competente, capaz de expressar suas necessidades, percepções e experiências, de forma que o cuidado em saúde seja planejado na sua perspectiva^(7)^.

Esperamos que as questões relacionadas à promoção da segurança de todos os neonatos e todas as crianças passem a integrar as agendas de prioridades de pesquisa nacionais, contribuindo para o fortalecimento do Sistema Único de Saúde e promovendo o desenvolvimento sustentável.

Em nosso país, a criança tem, como cidadã, o direito à assistência à saúde, e a sociedade tem como dever protegê-la. Como enfermeiros, temos o dever ético e moral de oferecer e promover assistência à saúde segura, livre de danos evitáveis, competente e comprometida com as necessidades das crianças, preservando sua dignidade e integridade, e garantindo o direito à “Segurança do Paciente desde o Início”.

# Translation: “Atención segura para cada recién nacido y cada niño”: La seguridad del paciente como prioridad desde el inicio

**DOI:** 10.1590/1518-8345.0000.4658

**Published:** 2025

**Authors:** Mavilde Luz Gonçalves Pedreira, Aline Santa Cruz Belela-Anacleto

**Affiliations:** 1 Universidade Federal de São Paulo, Escola Paulista de Enfermagem, São Paulo, SP, Brasil





Con el objetivo de sensibilizar y concienciar globalmente sobre la necesidad de reconocer, priorizar y abordar la vulnerabilidad de los niños ante los riesgos y daños asociados a la atención sanitaria insegura, la Organización Mundial de la Salud (OMS) anunció “Atención segura para cada recién nacido y cada niño” como tema del Día Mundial de la Seguridad del Paciente 2025, celebrado el 17 de septiembre - fecha clave en el calendario global de salud pública^(1)^.

La OMS insta a los países miembros a implementar políticas e intervenciones en áreas prioritarias para “aumentar la conciencia global sobre los riesgos en la atención pediátrica y neonatal en todos los entornos sanitarios, destacando las necesidades específicas de niños, familias y cuidadores; movilizar gobiernos, organizaciones de salud, entidades profesionales y la sociedad civil para adoptar estrategias sostenibles que garanticen una atención más segura, integrada en iniciativas más amplias de seguridad y calidad; empoderar a padres, cuidadores y niños mediante educación, concienciación y participación activa en el cuidado; y abogar por fortalecer la investigación en seguridad del paciente pediátrico y neonatal^(1)^.

Como enfermeras pediátricas, acogemos esta iniciativa con entusiasmo y responsabilidad, reconociendo su potencial para alinear la atención sanitaria con las demandas de seguridad de recién nacidos, niños, adolescentes y sus familias en nuestro país. La priorización de los niños en políticas de seguridad del paciente es imperativa debido a su vulnerabilidad y mayor riesgo de daños por eventos adversos - vinculados a factores demográficos, de desarrollo, dependencia de cuidadores y epidemiologías distintivas (los “4 D de la pediatría”)^(2)^.

Los niños son afectados desproporcionadamente por eventos adversos asociados a desventajas socioeconómicas, riesgos ambientales, falta de acceso a cuidados (especialmente para condiciones congénitas o críticas) y desigualdades por edad, raza, etnia y género^(2-3)^. Un ejemplo claro es la falta de medicamentos adecuados por edad, que eleva el riesgo de daños e incluso muertes por errores de medicación - un problema persistente desde el hogar hasta unidades de cuidados intensivos. A pesar de avances, la disponibilidad de formulaciones pediátricas y neonatales sigue siendo limitada, complicando la práctica y aumentando errores. En Brasil, a diferencia de otros países, la comercialización de formulaciones pediátricas (especialmente parenterales, usadas por décadas) no es obligatoria. Combatir esta inequidad requiere estudios específicos y/o incluir niños en ensayos clínicos.

La seguridad farmacológica es clave en pediatría. Aunque subestimados, los errores de medicación causan daños significativos. Los niños hospitalizados enfrentan tasas de eventos adversos hasta tres veces mayores que los adultos, con neonatos en riesgo particular. Factores como inmadurez fisiológica, cambios rápidos del desarrollo y la necesidad de dosificación individualizada (con cálculos complejos y variables en prescripción, preparación y administración) aumentan este riesgo^(4)^.

Expertos señalan que la seguridad farmacológica en pediatría ha sido descuidada. Urgen herramientas para monitorear eventos adversos en la práctica clínica, permitiendo estrategias de prevención. Paralelamente, es crucial usar tecnologías de apoyo a decisiones clínicas - para alergias, interacciones, dosificación y condiciones médicas. En neonatos, la investigación debe considerar sus necesidades éticas, prácticas y de bienestar únicas^(5)^.

El desarrollo infantil - un proceso dinámico y complejo - basa toda atención sanitaria orientada a su bienestar físico, mental, emocional y social. Así, la pediatría exige expertise en etapas de desarrollo y enfermedades diversas. Incluso en un mismo entorno, se requiere conocimiento integral - pero especializado - para manejar afecciones respiratorias, cardíacas, neurológicas, gastrointestinales, genitourinarias, dermatológicas, ortopédicas y más. La OMS resalta la seguridad diagnóstica: los errores o retrasos en diagnósticos pediátricos suelen causar daños graves o irreversibles. Además, la dependencia de los niños de los adultos - especialmente los más pequeños - añade complejidad: no pueden decidir, controlar tratamientos, cuestionar cuidados ni detallar su historial médico. Esto subraya la necesidad de profesionales capacitados para involucrar a las familias y educar a niños, adolescentes y cuidadores como agentes activos en su seguridad^(2-3)^.

Estudios globales demuestran que la cantidad y calificación de enfermeros impactan directamente en la mortalidad y morbilidad. El desequilibrio entre la demanda de cuidados y la oferta de enfermeros calificados compromete la seguridad y la confianza pública. En pediatría, la calificación enfermera - más que el número - reduce eventos adversos^(6)^. Su vulnerabilidad exige ratios más bajas; algunos estados de EE.UU., por ejemplo, requieren hasta cuatro niños por enfermero titulado en unidades clínicas/quirúrgicas y uno por cama en UCIs.

En Brasil, avanzar en seguridad pediátrica requiere políticas que mejoren tanto la cantidad como la formación de enfermeros. Los currículos de enfermería pediátrica varían ampliamente, tratando a veces la neonatología como especialidad en lugar de competencia esencial. Esto pone en riesgo a un grupo ya vulnerable y perpetúa injusticia social al no abordar el desarrollo infantil como parte integral de la formación en salud humana.

El progreso exige reconocer el derecho de los niños a participar en decisiones sobre su cuidado. La información debe compartirse según su edad y desarrollo, escuchando sus voces y protegiendo sus intereses. Este modelo los valora como agentes sociales activos, cuyas perspectivas deben moldear la atención^(7)^.

Esperamos que la seguridad de recién nacidos y niños se integre en agendas nacionales de investigación, fortaleciendo el Sistema Único de Salud (SUS) y promoviendo desarrollo sostenible.

Como ciudadanos, los niños tienen derecho a la salud, y la sociedad debe protegerlos. Como enfermeros, tenemos el deber ético de ofrecer atención segura, libre de daños evitables, competente y comprometida con sus necesidades - garantizando el derecho a “Seguridad del Paciente desde el Inicio”.
